# The Influence of Maturity Status on Resting Energy Expenditure, Body Composition and Blood Pressure in Physically Active Children

**DOI:** 10.3390/healthcare9020216

**Published:** 2021-02-16

**Authors:** Edyta Łuszczki, Maciej Kuchciak, Katarzyna Dereń, Anna Bartosiewicz

**Affiliations:** 1 Institute of Health Sciences, Medical College of Rzeszów University, 35-959 Rzeszów, Poland; kderen@ur.edu.pl (K.D.); abartosiewicz@ur.edu.pl (A.B.); 2 Institute of Physical Culture Sciences, Medical College of Rzeszów University, 35-959 Rzeszów, Poland; mkuchciak@ur.edu.pl

**Keywords:** children, maturation, physical activity, resting energy expenditure

## Abstract

Peak height velocity (PHV) is the period where the maximum rate of growth occurs. The moment the sports player reaches PHV can be estimated by monitoring the growth of body structures. The aim of this study was to assess changes in resting energy expenditure (REE), body composition and blood pressure in young, male soccer players between the pre-PHV, circa-PHV and post-PHV periods. This transverse study was conducted among 184 children aged 9 to 17 and included measurements of the resting energy expenditure (REE) using indirect calorimetry, body composition (bioimpedance) and blood pressure (sphygmomanometer). In addition, births in each quartile were analyzed. Children in the pre-PHV group had significantly lower REE values compared to the other two groups (*p* < 0.0001). The differences in the value of the REE between the children in the circum and post groups were not statistically significant. Additionally, the fat-free mass was significantly lower in the pre-PHV period compared to the other two periods (*p* < 0.0001), and the same relationship concerned the *z*-score body mass index (BMI) and systolic blood pressure. Early-born players were overrepresented (*p* < 0.05).

## 1. Introduction

Peak height velocity (PHV) is the period where the maximum rate of growth occurs. This is an indication of maturity, and it is one of the most used indicators in studies that investigate maturation processes in adolescents, right next to the Tanner staging [1,2,3]. The time when the sports player reaches PHV can be estimated by monitoring the growth of body structures (organs and limbs, as well as structures of the nervous, visual, auditory and musculoskeletal systems). Changes in body composition during adolescence are associated with the biological maturation state [4].

Young soccer players are grouped by their chronological age (CA) to reduce the effects of developmental differences. However, young athletes of the same chronological age can vary in their maturity status (stage of puberty, skeletal age, maturity timing) [5]. The literature on the subject indicates that youth soccer players of the same chronological age vary in their skeletal age by 5–6 tiers [6]. The advantage of being born early within a cohort has been termed the relative age effect (RAE) [7]. Individual differences in terms of maturity status are important for many sports disciplines as they have implications not only for development but also retention/exclusion. Sports players who are advanced in their maturity stage may have both functional and size advantages when compared with later-maturing youth athletes of the same chronological age [8]. As RAEs are based on chronological age, relatively older children consistently have an advantage due to their extended age, favoring advanced maturation [9]. Keeping track of biological maturation while assessing talent, along with the birth data cohort, may help prevent excluding possible talent that is missed because of birth dates in late trimesters and/or slower biological maturation.

Equations based on body mass, height, chronological age, sitting height and leg length can predict the maturity offset (time before or after PHV). Chronological age minus the predicted maturity offset gives a predicted age at PHV. Youth can be classified as pre-PHV, circa-PHV and post-PHV according to the predicted offset [1].

Monitoring body composition changes during puberty is very important because many components can influence body features in adults. Furthermore, it can be essential for young soccer players because these changes can influence their training, strength and power [10]. An adolescent’s growth is characterized by changes in body composition, especially those evolving in a sex-specific manner: fat and fat-free mass (FFM). An increase in FFM can be observed from five months to a year post-PHV, especially in boys [11], and this instance has been associated with implications in the development of strength and soccer-specific explosive movement [12].

Considering that FFM is the most metabolically active compound, an increase in FFM will also influence the resting energy expenditure (REE) [13]. 

Many cross-sectional and longitudinal studies have shown that REE increases during puberty [14]. Scientific evidence based on FFM-adjusted energy values is limited. Emphasis is mainly placed on the function of the rise in the level of hormones, such as insulin-like factor-1 (IGF-1), testosterone, growth hormone (GH) and changes in thyroid function [15]. Research on soccer players indicates that REE increases by about 400 kcal/day from the chronological ages of 10 to 13 [16]. Bidaurrazaga-Letona et al. examined the factors involved in the selection of young soccer players [17], and their results show that both body size and maturation are key elements. Another factor that can change during puberty is blood pressure (BP). BP increases as children become adolescents. During puberty, the rate of change in BP may accelerate [18,19]. A few studies have shown that increases in the body mass index (BMI) in adolescents may have an influence on BP [20], indicating that maturity status could be the main factor in the development of high BP during adulthood. 

In our study, we attempted an analysis of the relative age effect (RAE) issue in young soccer players. 

To the authors’ knowledge, no research has yet assessed the difference in REE between the pre-PHV, circa-PHV and post-PHV periods in youth soccer players. Therefore, the aims of this study were three-fold: to assess changes in (1) body composition, (2) blood pressure (3) and resting energy expenditure in youth male soccer players between the pre-PHV, circa-PHV and post-PHV periods.

## 2. Materials and Methods

### 2.1. Subjects

The inclusion criteria were: aged 9 to 18 years, male, ≥2 years of football training about 3 times/day and playing a match once a week and consent of the parent or guardian to participate in the study. The exclusion criteria were: no consent to participate in the study, illness or injury. The study was conducted in a randomly selected sports school during the 2018/2019 school year. In order to select the school, invitations were sent to the principals of all (7 schools) of the sports schools in the area (Podkarpackie Voivodeship, Poland). All of the schools represented a similar student profile and offered a similar sports program. Out of those that agreed (3 schools), one school was randomly selected by the cluster sampling method. A total of 276 parents/guardians of the children attending the school selected received an invitation letter, and 207 responses were received consenting to their child’s participation in the study. Of these responses, 23 participants were excluded from the study because they were not prepared for measurement. Finally, the study group consisted of 184 male children aged 9 to 17. 

Physical activity was measured on the basis of the School Sports Programme training, which involved: pitch-based training (technical and tactical soccer-specific training, with volume and intensities specific to the age group’s competitive soccer demands (~90 min/4 times per week)), gym-based training (movement competency exercises (~45 min/4 times per week)) and competitive match play (90 min/once per week). 

The study participants and their legal guardians received verbal and written information about the objectives, risks and benefits of the study. Both the guardians and the participants provided their informed written consent to participate in the study. 

### 2.2. Assessments

The research took place at the Laboratory for Innovative Research in Dietetics (Centre for Innovative Research in Medical and Natural Sciences, University of Rzeszów, Rzeszów, Poland). The rooms had a controlled temperature between 22 and 25 °C.

All recommendations concerning preparations for the study were outlined during the meeting with parents/guardians, including having rest, refraining from the consumption of meals 12 h before the measurement and refraining from drinking beverages with caffeine content 48 h before the measurement. Additionally, it included refraining from physical activity for the last 12 h. In the beginning, the subjects were informed of the entire test procedure, including the need to empty their bladders as required to minimize the risk of error during the body composition analysis. 

The method of conducting the examination was clarified in detail, and each participant had the chance to see the examination rooms with the equipment beforehand so that it did not raise concerns in the research group. 

#### 2.2.1. Anthropometric Measurements, Body Composition and Body Mass Index

Body height was measured at the beginning of the study visit with a Seca 213 portable stadiometer to the nearest 0.1 cm. The test participants were asked to remove their footwear and stand with their back to the stadiometer in an upright position during the measurement stage. The arithmetical average of three measurements was used for the analysis.

Sitting height was measured as the distance from the flat sitting surface to the top of the head with participants sitting in an upright position (head in the Frankfort horizontal plane), knees together and directed straight ahead. Sitting height was subtracted from height to provide an estimate of leg length.

Body composition was measured by the electrical bioimpedance method (6.25 kHz, 50 kHz, 90 µA) using a calibrated segment analyzer (Tanita MC-980 PLUS MA, Tokyo, Japan) with an accuracy of 0.1 kg/0.1%. In the literature, results involving children using the Tanita Analyzer are consistent with those obtained from dual-energy X-ray absorptiometry (DXA) [21]. The analyzer is equipped with 8 electrodes (4 built into the platform, the others are in holders). The participants stood on the analyzer without shoes, upright, with straight legs, making sure that the weight of their body was equally distributed between both feet. The participants held handles with electrodes, keeping their arms away from their body at an angle of 35°–40°. Body mass index (BMI) was calculated as body weight (kg)/height (m)^2^. Based on BMI values, the BMI *z*-score was also calculated. According to the World Health Organization (WHO) child growth standard, based on the WHO Reference 2007, children aged 6–19 years are overweight and obese with excess weight over 1 SD and 2 SD, respectively, and underweight under 2 SD [22]. Body fat (%), total body water content (kg) and fat-free mass (kg) were analyzed in the study.

#### 2.2.2. Arterial Blood Pressure

Blood pressure was measured three times according to the recommendations of the National High Blood Pressure Education Program Working Group in Children and Adolescents (NHBPEP) [23], using a Welch Allyn 4200B-E2 blood pressure meter (Aston Abbotts, UK) with cuffs sized to fit the participants’ arm circumference. The arithmetical average of three measurements was calculated for each subject tested. Systolic and diastolic pressure values were used for the analysis.

#### 2.2.3. Resting Energy Expenditure

REE was measured by means of the indirect calorimetry method using an indirect calorimeter (Fitmate MED, COSMED, Rome, Italy). The indirect calorimeter was calibrated according to the manufacturer’s instructions with a 3 L syringe (COSMED; P/N: C00600-01-11). REE was examined using silicone face masks for the children (a petite/pediatric size COSMED, Rome, Italy). The children were asked to lay on their back with a pillow under their head on a couch (Norma II, Juventus, Poland). All participants were instructed to lie still, avoid speaking and not fall asleep during the test. A turbine flowmeter was used to measure ventilation, and the analysis of exhaled oxygen was possible due to the presence of a galvanic cell sensor. The ratio of CO_2_ produced to O_2_ determines the respiratory quotient (RQ). VCO_2_ is not measured directly; rather, it is estimated with a constant respiratory rate (RQ) of 0.85. Fitmate monitors oxygen uptake (VO_2_), fraction of O_2_ expired (FeO_2_), ventilation (Ve), heart rate (HR) and respiration rate (Rf). Estimation of REE (kcal/d) is possible thanks to the modified Weir equation: REE = [5.675 × VO_2_ + 1.593 × VCO_2_ − 21.7], where VO_2_ is the oxygen volume in the breath (mL/min), and VCO_2_ is the carbon dioxide output (mL/min) [24]. The Fitmate Med calorimeter was validated, and high reliability of the measurements was obtained [25]. The results obtained from the Fitmate Med device are comparable to the Douglas bag system, which uses a sensor to measure VCO_2_ [26]. 

#### 2.2.4. Age at Peak Height Velocity (PHV)

Somatic maturity was estimated for each participant by calculating the maturity offset with the equation proposed by Mirwald et al. [1]: 

Maturity Offset

= −9.236 + (0.0002708 × [Leg Length × Sitting Height])

+ (0.001663 × [CA × Leg Length])

+ (0.007216 × [CA × Sitting Height])

+ (0.02292 × [Weight by Height Ratio × 100]).

The equation calculates the time in years from PHV and includes: chronological age, height, sitting height and weight. The equations are accurate to ±0.24 years [9]. A maturity-offset value was estimated for youth players aged 9–17 as this is characteristically the timeframe in which PHV occurs in youth soccer players [27]. In addition, the equation was developed in this age range [1]. CA at prediction minus maturity offset provided a predicted age at PHV (years).

##### PHV Periods

To allow for the comparison of REE, body composition and blood pressure in different PHV periods, categorization of the individual as pre-PHV (offset < −1 years), circa-PHV (≤±1 years), or post-PHV (offset > +1 years) has been obtained [28].

### 2.3. Statistical Analysis

The study results were obtained using descriptive statistics: number (*n*), standard deviation (SD) and standard error (SE). 

The Shapiro–Wilk test was performed to check the normality of the data. One-way analysis of variance (ANOVA) was used to compare the means of two or more samples (the PHV groups and the selected dependent variable) using the F distribution. Additionally, Tukey’s honestly significant difference (HSD) test was used to test the differences among the sample means for significance. For each PHV group, the median of the reported values for the individual studies was calculated along with the quartile limits. A chi-square test was used to test the statistical difference between the observed and expected birth distributions with the percentage of births in each quartile. Statistical significance was established as *p* < 0.05. Calculations were performed with the Statistica 10.0 tool (StatSoft, Inc., Tulsa, Oklahoma, OK, USA).

### 2.4. Ethics

This research project was carried out in accordance with the Declaration of Helsinki. The study was approved by the institutional Bioethics Committee at the University of Rzeszów (Resolution No. 2/01/2019) and all appropriate administrative bodies.

## 3. Results

### 3.1. Characteristics of the Study Group

A total of 184 boys aged 9 to 17 years participated in the study. The mean age of the respondents was 13.07 ± 2.23 years. Table 1 shows the results of the descriptive statistics for the individual PHV groups for the selected variables. Participants have been divided into three groups: pre, circum and post. In the pre-PHV group, there were 64 boys, in the circum 48 and in the post-PHV group 72 children.

Average physical activity was 10.69 ± 0.59 h/week for each participant (median = 10.50 h/week, minimum = 10 h/week, maximum = 12 h/week).

Our results allow analyzing the differences between the chronological age and the age at PHV. It was observed that in the pre-PHV group, the age at PHV was higher than the chronological age in the same group. The main reason for this could be that there was an overabundance of first-semester birth months in this group. When analyzing the births in each quartile, a skewed distribution was observed toward the first quartile in both the total sample and in each age group compared with the second, third and fourth quartiles. Early-born players were overrepresented (*p* < 0.05). The opposite effect was shown in the post-PHV group, where age at PHV was lower than CA (Table 1). In addition, we observed a greater effect of measured variables between the pre- and post-PHV groups, especially in REE, FFM, total body water (TBW) content and systolic pressure.

### 3.2. The Findings

The values of the individual parameters depending on the PHV periods are presented in Table 2. There were statistically significant differences (F = 40.155; *p* < 0.0001) between the REE values among the children in the three analyzed groups: pre (1528.33 ± 191.29), circum (1858.87 ± 233.97) and post (1939.97 ± 323.88). When examining pairwise comparisons, it was found that the children in the pre group had significantly lower REE levels compared to the other two groups (*p* < 0.0001). The differences in the value of the REE between the children in the circum and post groups were not statistically significant (*p* = 0.2804). A similar observation was obtained when analyzing the REE/kg body weight (BW). However, when comparing the PHV groups and the REE/kg FFM, significant results were observed only between the pre- and post-PHV groups. Participants in the pre-PHV group had a significantly lower RRE/kg FFM ratio than children in the post-PHV group (*p* = 0.020).

Statistically significant differences were also shown for the *z*-score BMI (*p* < 0.0001). Children in the pre-PHV group had a significantly lower *z*-score BMI than the circum and post groups (*p* < 0.0001). The difference between the children in the circum and post groups was also significantly lower (*p* = 0.0062).

When examining differences in body composition, no differences in body fat were found between the groups (*p* = 0.3669). However, the fat-free mass was significantly lower in the pre-PHV period compared to the other two periods (*p* < 0.0001), and between the children in the circum and post groups it was not statistically significant (*p* = 0.0648). In addition, total body water content in the children in the pre-PHV group was significantly lower than the circum and post groups, and total body water (TBW) content between the children in the circum and post groups was not statistically significant (*p* = 0.0568).

When examining the differences in blood pressure, no significant differences in diastolic pressure between the groups were observed (*p* = 0.5557). However, systolic pressure was significantly different between the groups (F = 4.561; *p* = 0.0118). When comparing in pairs, significant differences were shown for the pre and post groups. Children in the pre-PHV group had significantly lower systolic pressure than children in the post-PHV group (*p* = 0.0095).

## 4. Discussion

Relative age effects (RAEs) refer to age differences between children in the same selection year. Our results in this study suggest that RAE can exist in Polish soccer players. Month of birth and maturity status are variables that produce this effect. The organization of youth soccer into age groups based on the calendar year may lead to one- or two-year differences between the players. Our results show the differences between the chronological age and the age at PHV and should encourage coaches and medical staff to evaluate the maturity status of players in categories such as the PHV to achieve a view of players’ potential and performance. Observing biological maturation while assessing talent along with the birth data cohort may help prevent excluding possible talent that is missed because of birth dates in late trimesters. It is possible that early-born players have an advantage. An unbalanced distribution was reported among players born in the first, second, third, and fourth quartiles, favoring those players born earlier in the selection year. These results are similar to previous research analyzing RAE [29,30].

As demonstrated by our results, the REE values among children from the three analyzed groups—pre (1528.33 ± 191.29), circum (1858.87 ± 233.97) and post (1939.97 ± 323.88)—were statistically significantly different. Children in the pre-PHV period had significantly lower REE levels compared with the other two groups (*p* < 0.0001). The differences in the value of the REE between the children from the circum and post groups were not statistically significant (*p* = 0.2804). These results are in agreement with the data of a previous study on Indian soccer players, where an increase in REE of about 400 kcal/day from the ages of 10 to 13 was also noticed [31]. A similar effect was observed by Hannon et al., who also showed an increase in REE between the U12 and U14 (Football Academy Categories) age groups [32].

The state of maturity status affected the BMI and body composition of the youth soccer players. The statistical analysis presented differences in most of the measured variables, mainly in the pre-PHV group compared with the post-PHV group. When it comes to height and weight, the same tendency was noticed; that is, it increased in older children. Jorquera et al. presented differences in height and weight between the U-16 and U-17 categories among soccer players [33]. All things considered, these factors could contribute to the increment or the decrease in reaching the PHV. On the other hand, no differences in body fat were found between the groups (*p* = 0.3669). This may mean that body fat content is independent of the PHV period. However, the fat-free mass was significantly lower in the pre-PHV period compared to the other two periods (*p* < 0.0001), but between the children of the circum and post groups it was not statistically significant (*p* = 0.0648). 

In their study, Gil et al. examined anthropometry among soccer players who were born in the same year. They analyzed the differences between the older and younger athletes. Their results showed that older players were taller and presented a higher fat-free mass. Similarly, they manifested better performance [34]. As was presented in the literature on the subject, the growth acceleration should be taken into consideration by coaches, especially when they are practicing with young athletes. More mature soccer players could present more developed anthropometric parameters [35].

Biological, behavioral and social variables have been connected to BP in childhood and adolescence [36,37]. The natural increase in BP values with an improvement in chronological age is related to weight, height and changes in body composition [38]. The results of the research also showed that when examining the differences in blood pressure between the PHV periods, no significant differences in diastolic pressure were observed (*p* = 0.5557). However, the systolic pressure was significant (F = 4.561; *p* = 0.0118) in the study group. Children in the pre-PHV group had significantly lower systolic pressure than children in the post-PHV group. In a recent study, researchers observed that increases in systolic blood pressure were much greater during the pubertal growth period than before or after. The rates of increase in systolic BP during the pubertal growth period was 3–6 times larger than those in the prepubertal growth period. The rise in BP during pubertal growth is probably connected to the increase in gonadal hormones, and other hormones could also have an influence, such as the growth hormone (GH) [39]. 

### Limitations

As this study was cross-sectional, causality and temporality issues should not be considered. In addition, only one school was involved in the study, which may also lead to selection bias. Further studies should include more schools as well as other sports disciplines. Assessment of body composition by the electrical impedance method is another limitation. Although this method is used in studies by many researchers, measuring body composition using DXA would allow for more accurate results. In the study, a portable indirect calorimeter was used. Fitmate Med using a constant RQ may introduce differences in the estimate of oxygen consumption and REE values. This may lead to errors when compared with the gold standard. Fitmate has been previously validated as a suitable alternative to the traditional indirect calorimetry by previous studies. Despite not measuring CO_2_ production, it is very convenient in the clinical setting, assuming a minimal error of analysis. Estimating the PHV by using anthropometric-equation-based methods may cause limitations as an indicator of maturity timing. It will be better to use radiographic technology, which could decrease internal limitations, in predicting PHV. Furthermore, the Tanner staging is the most common method of measuring maturity.

## 5. Conclusions 

Peak height velocity is a significant aspect of body development among young soccer players. It is worth mentioning that maturity status is strongly related to anthropometric components, resting energy expenditure and body composition. Therefore, it would be very useful to take maturity status into consideration when coaches train and medical services of soccer teams work with young athletes.

## Figures and Tables

**Table 1:** Descriptive statistics for the individual peak height velocity (PHV) groups for the selected variables.

Variables PHV Group		Mean	SD	SE		95% CI		Min	Max
						Lower Limit	Upper Limit		
REE (kcal/day) *	Pre	1528.33	191.287	25.337	1477.58	1579.09	1187	2054	
	Circum	1858.87	233.970	37.465	1783.03	1934.72	1317	2377	
	Post	1939.97	323.883	40.173	1859.71	2020.22	1219	2604	
	Total	1774.59	319.710	25.197	1724.83	1824.35	1187	2604	
REE/kg BW	Pre	41.60	6.044	0.801	40.00	43.21	26.87	56.68	
	Circum	35.86	4.978	0.797	34.24	37.47	22.88	46.29	
	Post	31.08	3.871	0.480	30.12	32.04	23.44	40.87	
	Total	35.96	6.759	0.533	34.91	37.01	22.88	56.68	
REE/kg FFM	Pre	35.19	6.920	0.917	33.35	37.06	21.23	52.06	
	Circum	38.65	6.809	1.090	36.44	40.85	21.23	52.06	
	Post	36.61	4.781	0.593	35.43	37.80	25.11	46.83	
	Total	36.60	6.217	0.490	35.63	37.57	21.23	55.72	
Age (years)	Pre	10.59	1.060	0.139	10.31	10.86	9	12	
	Circum	13.21	1.025	0.158	12.89	13.53	12	15	
	Post	15.17	1.001	0.123	14.92	15.41	13	16	
	Total	13.07	2.231	0.173	12.73	13.41	9	16	
Height (cm)	Pre	145.93	7.793	1.023	143.88	147.98	127	159	
	Circum	163.76	9.066	1.399	160.94	166.59	145	179	
	Post	173.06	8.225	1.012	171.04	175.08	154	191	
	Total	161.23	14.420	1.119	159.02	163.44	127	191	
Weight (kg)	Pre	37.43	7.241	0.951	35.53	39.33	25	57	
	Circum	52.55	9.590	1.480	49.56	55.54	34	77	
	Post	62.67	9.776	1.203	60.26	65.07	44	102	
	Total	51.29	14.082	1.093	49.13	53.45	25	102	
BMI (*z*-score)	Pre	−0.79	0.82	0.11	−1.01	−0.58	−2.26	1.70	
	Circum	0.02	1.02	0.16	−0.29	0.34	−1.49	3.68	
	Post	0.55	0.80	0.10	0.36	0.75	−0.94	3.33	
	Total	−0.05	1.04	0.08	−0.21	0.11	−2.26	3.68	
Fat (%)	Pre	18.838	4.7142	0.6190	17.598	20.077	13.0	34.5	
	Circum	17.781	4.7276	0.7295	16.308	19.254	8.8	28.6	
	Post	17.771	4.4043	0.5421	16.689	18.854	13.0	30.0	
	Total	18.146	4.5968	0.3568	17.442	18.851	8.8	34.5	
FFM (kg)	Pre	44.864	8.2034	1.0772	42.707	47.021	30.0	61.6	
	Circum	49.514	9.3073	1.4361	46.614	52.415	30.0	79.6	
	Post	53.285	8.1128	0.9986	51.290	55.279	35.0	80.0	
	Total	49.389	9.1638	0.7112	47.984	50.793	30.0	80.0	
TBW (kg)	Pre	32.841	6.0059	0.7886	31.262	34.421	22.0	45.1	
	Circum	36.231	6.7772	1.0458	34.119	38.343	22.0	58.3	
	Post	39.059	5.9668	0.7345	37.592	40.526	26.0	58.0	
	Total	36.171	6.7182	0.5214	35.142	37.201	22.0	58.3	
Systolic pressure (mmHg)	Pre	113.66	12.573	1.651	110.35	116.96	91	150	
	Circum	115.88	11.443	1.766	112.32	119.45	92	140	
	Post	120.00	11.484	1.414	117.18	122.82	99	142	
	Total	116.74	12.120	0.941	114.88	118.60	91	150	
Diastolic pressure (mmHg)	Pre	66.26	6.493	0.853	64.55	67.97	54	83	
	Circum	65.07	6.414	0.990	63.07	67.07	50	79	
	Post	65.12	6.678	0.822	63.48	66.76	50	80	
	Total	65.51	6.532	0.507	64.51	66.51	50	83	
Age (PHV)	Pre	13.621	0.604	0.079	13.462	13.779	12.1	15.0	
	Circum	13.783	0.833	0.129	13.524	14.043	11.3	15.3	
	Post	13.430	0.777	0.096	13.239	13.621	11.7	15.1	
	Total	13.586	0.746	0.058	13.472	13.700	11.3	15.3	
Maturity offset (years)	Pre	−2.557	0.816	0.107	−2.772	−2.342	−3.9	−1.2	
	Circum	−0.067	0.745	0.115	−0.299	0.165	−1.0	1.0	
	Post	2.279	0.730	0.090	2.099	2.458	1.2	3.8	
	Total	−0.004	2.226	0.173	−0.345	0.337	−3.9	3.8	

* The respiratory ratio (R) during the study was 0.85. Me—median, SD—standard deviation, SE—standard error, Min—sample minimum, Max—sample maximum, CI—confidence interval, REE—resting metabolic rate, BMI—body mass index, FFM—fat-free mass, TBW—total body water, PHV—peak height velocity, BW—body weight.

**Table 2:** Values of individual parameters depending on the PHV periods (multiple comparisons).

Dependent PHV Variables Group			MD	SE	*p*	95% CI		F	*p*
						Lower Limit	Upper Limit		
REE (kcal/day)	Pre	Circum	−330.538	54.439	<0.0001	−459.34	−201.74	40.155	<0.0001
		Post	−411.636	47.537	<0.0001	−524.11	−299.16		
	Circum	Pre	330.538	54.439	<0.0001	201.74	459.34		
		Post	−81.097	53.061	0.2804	−206.64	44.44		
	Post	Pre	411.636	47.537	<0.0001	299.16	524.11		
		Circum	81.097	53.061	0.2804	−44.44	206.64		
REE/kg BW	Pre	Circum	5.748	1.039	<0.0001	3.29	8.21	67.332	<0.0001
		Post	10.523	0.907	<0.0001	8.38	12.67		
	Circum	Pre	−5.748	1.039	<0.0001	−8.21	−3.29		
		Post	4.775	1.012	<0.0001	2.38	7.17		
	Post	Pre	−10.523	0.907	<0.0001	−12.67	−8.38		
		Circum	−4.775	1.012	<0.0001	−7.17	−2.38		
REE/kg FFM	Pre	Circum	−3.458	1.271	0.020	−6.46	−0.45	3.704	0.027
		Post	−1.421	1.110	0.408	−4.05	1.20		
	Circum	Pre	3.458	1.271	0.020	0.45	6.46		
		Post	2.037	1.238	0.230	−0.89	4.97		
	Post	Pre	1.421	1.110	0.408	−1.20	4.05		
		Circum	−2.037	1.238	0.230	−4.97	0.89		
Height (cm)	Pre	Circum	−17.831	1.682	<0.0001	−21.81	−13.85	167.529	<0.0001
		Post	−27.130	1.494	<0.0001	−30.66	−23.60		
	Circum	Pre	17.831	1.682	<0.0001	13.85	21.81		
		Post	−9.299	1.638	<0.0001	−13.17	−5.42		
	Post	Pre	27.130	1.494	<0.0001	23.60	30.66		
		Circum	9.299	1.638	<0.0001	5.42	13.17		
Weight (kg)	Pre	Circum	−15.117	1.807	<0.0001	−19.39	−10.84	124.084	<0.0001
		Post	−25.236	1.606	<0.0001	−29.03	−21.44		
	Circum	Pre	15.117	1.807	<0.0001	10.84	19.39		
		Post	−10.119	1.761	<0.0001	−14.28	−5.95		
	Post	Pre	25.236	1.606	<0.0001	21.44	29.03		
		Circum	10.119	1.761	<0.0001	5.95	14.28		
BMI (*z*-score)	Pre	Circum	−0.8138	0.1753	<0.0001	−1.2283	−0.3993	37.499	<0.0001
		Post	−1.3446	0.1557	<0.0001	−1.7128	−0.9764		
	Circum	Pre	0.8138	0.1753	<0.0001	0.3993	1.2283		
		Post	−0.5308	0.1707	0.0062	−0.9346	−0.1269		
	Post	Pre	1.3446	0.1557	<0.0001	0.9764	1.7128		
		Circum	0.5308	0.1707	0.0062	0.1269	0.9346		
Fat %	Pre	Circum	1.0570	0.9313	0.4940	−1.146	3.260	1.009	0.3669
		Post	1.0667	0.8273	0.4032	−0.890	3.024		
	Circum	Pre	−1.0570	0.9313	0.4940	−3.260	1.146		
		Post	0.0097	0.9073	0.9999	−2.136	2.156		
	Post	Pre	−1.0667	0.8273	0.4032	−3.024	0.890		
		Circum	−0.0097	0.9073	0.9999	−2.156	2.136		
FFM (kg)	Pre	Circum	−4.6505	1.7140	0.0201	−8.705	−0.596	15.301	<0.0001
		Post	−8.4211	1.5226	<0.0001	−12.022	−4.820		
	Circum	Pre	4.6505	1.7140	0.0201	0.596	8.705		
		Post	−3.7706	1.6698	0.0648	−7.720	0.179		
	Post	Pre	8.4211	1.5226	<0.0001	4.820	12.022		
		Circum	3.7706	1.6698	0.0648	−0.179	7.720		
TBW (kg)	Pre	Circum	−3.3896	1.2549	0.0208	−6.358	−0.421	15.557	<0.0001
		Post	−6.2177	1.1148	<0.0001	−8.855	−3.581		
	Circum	Pre	3.3896	1.2549	0.0208	0.421	6.358		
		Post	−2.8281	1.2226	0.0568	−5.720	0.064		
	Post	Pre	6.2177	1.1148	<0.0001	3.581	8.855		
		Circum	2.8281	1.2226	0.0568	−0.064	5.720		
Systolic pressure (mmHg)	Pre	Circum	−2.226	2.404	0.6248	−7.91	3.46	4.561	0.0118
		Post	−6.345	2.136	0.0095	−11.40	−1.29		
	Circum	Pre	2.226	2.404	0.6248	−3.46	7.91		
		Post	−4.119	2.342	0.1869	−9.66	1.42		
	Post	Pre	6.345	2.136	0.0095	1.29	11.40		
		Circum	4.119	2.342	0.1869	−1.42	9.66		
Diastolic pressure (mmHg)	Pre	Circum	1.187	1.327	0.6443	−1.95	4.33	0.590	0.5557
		Post	1.137	1.179	0.5999	−1.65	3.92		
	Circum	Pre	−1.187	1.327	0.6443	−4.33	1.95		
		Post	−0.050	1.292	0.9992	−3.11	3.01		
	Post	Pre	−1.137	1.179	0.5999	−3.92	1.65		
		Circum	0.050	1.292	0.9992	−3.01	3.11		

Me—median, MD—mean difference, SD—standard deviation, SE—standard error, CI—confidence interval, REE—resting metabolic rate, BMI—body mass index, FFM—fat-free mass, TBW—total body water, BW—body weight, F—F-test result; *p*—*p*-value, indicate significant values (*p* < 0.05).

## Data Availability

The data presented in this study are available on reasonable request from the corresponding author: eluszczki@ur.edu.pl.
